# Is It Me or the Robot? A Critical Evaluation of Human Affective State Recognition in a Cognitive Task

**DOI:** 10.3389/fnbot.2022.882483

**Published:** 2022-08-01

**Authors:** Doreen Jirak, Motonobu Aoki, Takura Yanagi, Atsushi Takamatsu, Stephane Bouet, Tomohiro Yamamura, Giulio Sandini, Francesco Rea

**Affiliations:** ^1^Robotics, Brain and Cognitive Science Group (RBCS), Istituto Italiano di Tecnologia, Genova, Italy; ^2^Department of Computer Science, Bioengineering, Robotics and Systems Engineering, University of Genoa, Genova, Italy; ^3^Mobility and AI Laboratory, Research Division, Nissan Motor Co., Ltd., Atsugi, Japan

**Keywords:** human-robot interaction, social robots, cognitive load, affective states, action units

## Abstract

A key goal in human-robot interaction (HRI) is to design scenarios between humanoid robots and humans such that the interaction is perceived as collaborative and natural, yet safe and comfortable for the human. Human skills like verbal and non-verbal communication are essential elements as humans tend to attribute social behaviors to robots. However, aspects like the uncanny valley and different technical affinity levels can impede the success of HRI scenarios, which has consequences on the establishment of long-term interaction qualities like trust and rapport. In the present study, we investigate the impact of a humanoid robot on human emotional responses during the performance of a cognitively demanding task. We set up three different conditions for the robot with increasing levels of social cue expressions in a between-group study design. For the analysis of emotions, we consider the eye gaze behavior, arousal-valence for affective states, and the detection of action units. Our analysis reveals that the participants display a high tendency toward positive emotions in presence of a robot with clear social skills compared to other conditions, where we show how emotions occur only at task onset. Our study also shows how different expression levels influence the analysis of the robots' role in HRI. Finally, we critically discuss the current trend of automatized emotion or affective state recognition in HRI and demonstrate issues that have direct consequences on the interpretation and, therefore, claims about human emotions in HRI studies.

## 1. Motivation and Related Work

The field of human-robot interaction (HRI) has manifold facets from robotic design to safe and intuitive collaborations between the robot and the human. The ultimate goal of HRI is to embed robotic agents naturally in the human environment to facilitate everyday life in domestic environments, health care, and education. Fundamental questions of how to implement important insights from psychology, social, and cognitive sciences for positively perceived interactions culminated in the research area of social robotics. In this area, a robot is not only assumed to perform monotone tasks as seen in industry applications but elicits so-called social cues or social signals (Poggi and D'Errico, 2012) and is physically embodied in the interaction with a human (Wainer et al., 2006). Important cues are the eye gaze (Admoni and Scassellati, 2017) or joint attention (Tan et al., 2020; Stephenson et al., 2021), and verbal communication to initiate task engagement (Castellano et al., 2012). Other significant behaviors include gestures (Barros et al., 2014), head movements (e.g., nodding) (McGinn, 2020; a recent survey on nonverbal communication in HRI; Clark and Ahmad, 2021) and facial expressions such as smiling. A robot displaying social cues has a fundamental impact on its anthropomorphic design and its humane appearance. Studies have shown that a “human-like” robot increases its acceptance as a valid HRI partner (Fink, 2012) correlated with a higher agreement concerning perceived intelligence and likability (Salem et al., 2013; Hoffmann et al., 2020). Furthermore, the anthropomorphism and display of social cues positively influence a human's trust in a robot (Gaudiello et al., 2016; Natarajan and Gombolay, 2020; Babel et al., 2021; a recent survey by Naneva et al., 2020). Moreover, socially-behaving robots can boost task performance in collaboration with Vasalya et al. (2018) or cognitive tasks like the Stroop test (Spatola et al., 2019) or the Eriksen flanker test (Spatola et al., 2020)^1^ (recent meta-analysis is provided by Roesler et al., 2021). However, experimental settings between a human and a socially-behaving, human-like robot in HRI are prone to fall into the uncanny valley (Mori, 1970; Laakasuo et al., 2021). Humans also tend to project expectations on a social robot (Ghiglino et al., 2020) tied with an overestimation of their actual skills. Failure e.g., natural dialogue negatively affects prospect interactions (Schramm et al., 2020). Moreover, HRI research showed that the attribution of personality traits like extroversion or dominance to the robot can serve as predictors of their social acceptance (Woods et al., 2005; Mileounis et al., 2015; Dou et al., 2019; Tanevska et al., 2019; Mou et al., 2020). Based on the similarity-complementary hypothesis, it remains inconclusive whether humans do prefer robots with similar personality characteristics or not (Esterwood et al., 2021) and whether the personality preference is task-dependent (Joosse et al., 2013).

Human behaviors, when exposed to robot platforms in everyday life situation, has been usually evaluated using standard statistical tools and questionnaires as well as human behaviors such as eye gaze patterns, blink frequency, and reaction times. Also, multimodal approaches which include physiological signals like changes in the electrodermal activity and heart rates are applied. Although these tools allow the identification of affective-related responses such as stress, e.g., increased blinking, the usage of external sensor devices for eye tracking or skin conductance can impede a clean analysis. Additionally, statistical assumptions like normally distributed data or linear relationships as in regression models may not hold. Therefore, it is desirable to extract critical human affective features automatically from intuitive interfaces like cameras. In the last years, the release of publicly open huge face databases (Guo et al., 2016) together with the facilitated usage of deep learning architectures thanks to GPU computing has laid the foundation to a new research are called “Affective Computing”. Researchers in this field work at the interdisciplinary border of social and developmental psychology, neuro, and computer science and develop computational models capturing human affective states and emotional responses. For HRI, the analysis of human affective states allows further insight into the level of anthropomorphism in humanoid robots to avoid the uncanny valley effect. It helps to shape interaction scenarios where negative emotional states like frustration or stress can be detected online, giving a robot the possibility to intervene immediately by displaying a smile or increasing the distance to the human to create a better comfort zone. Affective state and emotional facial expression recognition are also vital to foster HRI toward trust (Gaudiello et al., 2016), engagement (Kompatsiari et al., 2017; Babel et al., 2021), and rapport (Hoffmann et al., 2020), targeting long-term relationships between a human and a robot. The most prominent features in HRI for detecting affective states or emotions in facial expressions are arousal and valence (McColl et al., 2016), and so-called action units (Liu and Kappas, 2018; van Eijndhoven et al., 2020).

Our study extends a recent study by Aoki et al. (2022), which explored the effects on humans performance given a cognitively demanding task (MATB) in two conditions, i.e., in presence of a social vs. a non-social robot. As a robotic platform, we chose the humanoid icub robot, whose appearance resembles a child of around 3 years. The icub robot is equipped with a full body and has sensors such as a camera, and microphone/speaker, and can display facial expressions *via* LEDs installed into the icub head. A full overview of the icub robot platform is provided in Metta et al. (2008) and references therein. The behaviors shown by the icub robot during the experiments featured gestures and body movement, either with or without meaning (e.g., pointing gesture vs. random arm movement), speech parts at the start and the end (additionally after a task onset in the *social* condition) and display of emotions in the icub robot face. We found evidence that the presence of a social robot positively affects task performance supported by a reduced completion time and mental workload. Additionally, the participants attributed higher intelligence to the socially-behaving robot and reported to have enjoyed the interaction more (Aoki et al., 2022).

In this study, we build on our recent study and evaluate the progress of affective states and emotions elicited by facial expressions of humans performing the MATB task in three different conditions: the baseline which is called “*no-robot*,” a “*non-social*” condition where the robot expresses only behaviors that do not have social meaning, and a “*social*” condition, where the robot elicits some social attitude through very specific communicative social behavior typical of human-human interaction. Our analysis is based on multi-modal features such as the arousal-valence, gaze patterns, and detection of action units (AU). We hypothesize that the human participants show higher levels of positive affective states (arousal-valence) and emotions (action units) as well as least stress (gaze, eye blinking) in the presence of a social robot compared to the other two conditions. Also, we consider humans in the *no-robot* condition to display the least emotional responses, yielding a rather monotone pattern of facial signals. Given the increased usage of publicly available feature extraction tools, we also discuss the limitations of current automatic facial expression and emotion recognition as their vulnerability to environmental or technical factors has a huge impact on the interpretation of affective states and emotions in HRI.

## 2. Experimental Tasks and Conditions

Our research aims at exploring the affective states of the human partner when performing a demanding cognitive task when in presence of an assisting robot. Therefore, we selected the Multi-Attribute Task Battery (MATB), initially developed by NASA (Santiago-Espada et al., 2011). In brief, the task consists of three events that demand the participants' response and has a total length of 5 min:

1. A system monitoring task: the participant is asked to correct the changing colors of six different lights presented on the computer monitor by pressing a key (F1–F6 on the computer keyboard). The colors change sequentially, i.e., there is only one key-pressing event per time.
2. A tracking task: tracking occurs throughout the course of the experiment and demands the participant to correct *via* a joystick a moving object into its predefined, square area displayed on the monitor.
3. A communications task: the participant receives a voice command asking to change a specific radio frequency. The task is accomplished by pressing the arrow-keys on the computer keyboard.

**Figure 1:**
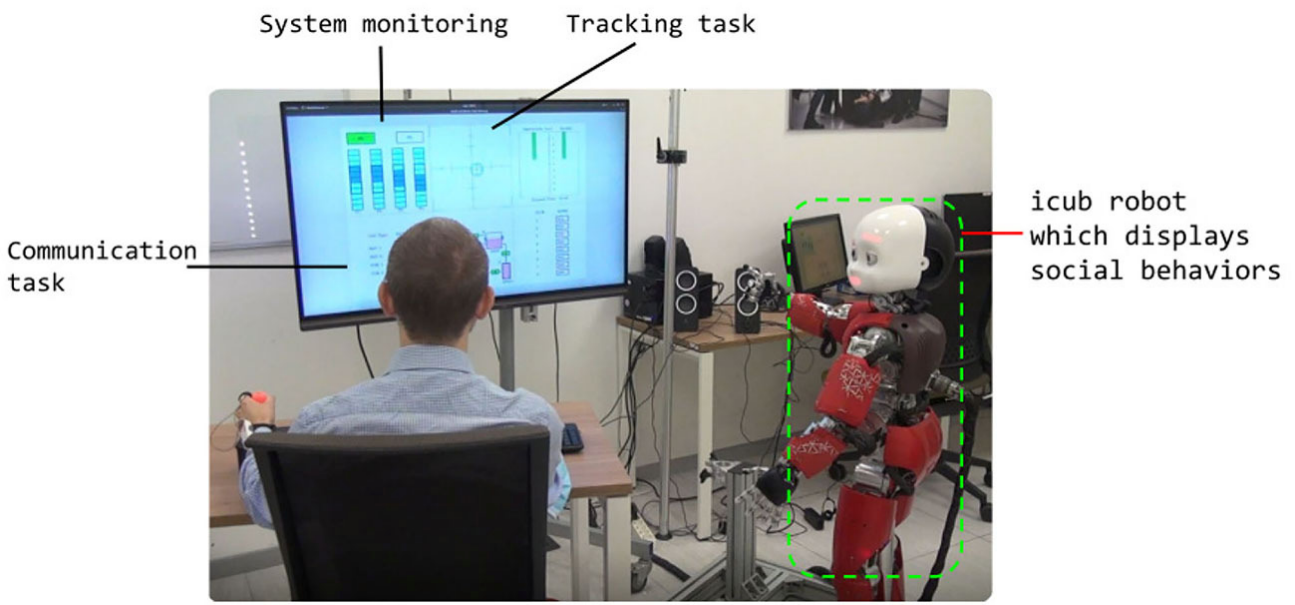
The experimental set up: a participant is seated in front of a computer monitor which displays the different subtasks included in MATB. The participant executes the different duties *via* keyboard or joystick and their face and upper body are recorded using a web camera. Depending on the condition, the icub robot is present throughout the course of the experiment. The robot's face expressions are controlled *via* LEDs, other body movements are predefined (Adapted from Aoki et al., 2022).

Figure 1 shows the entire experimental set up, which was approved by the Liguria Regions' local ethical committee in Italy (n. 222REG2015). For all three conditions, we recruited 15 participants who were assigned to only one of the conditions (between-subject design). All participants gave their informed consent including the recording of their face and upper body. Before the experiment, each participant received instructions and explanations from the experimenter. Moreover, each experiment started with a calibration phase for the joystick. Also shown in Figure 1 is the humanoid robot “icub” (Metta et al., 2008, 2010; Parmiggiani et al., 2012; Fischer et al., 2018). Its design resembles human face attributes and it features speech and motor behaviors, which is why the icub robot has been employed in numerous HRI and social robotics studies (Anzalone et al., 2015; Li, 2015).

We defined three different conditions with varying interaction levels of the icub robot and the expression of social cues.

- The *no-robot* condition consists of the execution of the MATB exercise by the participant without the presence of the iCub robot (baseline condition).
- In the *non-social* condition, the icub robot is present during the whole experiment and interacts at predefined time instances with the human. The interaction includes verbal output from the icub robot that does not carry social meaning (social neutral behavior), e.g., “Press key F1.” For the body movement of the robot, we aimed at a rather mechanistic behavior that does not communicate any social intention. As an example, an arm movement does not represent a pointing gesture but rather a movement without communicative meaning. For the communicative vocal behavior, neither modulation of the voice was used nor additional words following an etiquette like “please” or emotional facial expressions like smiling.
- The integration of socially-accepted norms^2^ was implemented in the *social* condition. Similar to the *non-social* condition, we defined specific interaction timing and created the verbal output as well as the facial expressions and motor behavior adding very specific social cues studied in human-human interaction. For instance, the icub robot greeted each participant similar to an experimenter. Moreover, the robot displayed emotional facial expressions like smiling and added words like “please” and “thank you” to the commands. After the task completion, the icub robot says goodbye.

The iCub robot was positioned in order to stimulate the peripheral sight of the participant. Furthermore, the number of body movements involved in the pointing (affecting participant's visual attention) and verbal communication involved in task support (affecting participant‘s auditory attention) on the robot are designed to limit the attentional drawing from the MATB task. Both the time onsets of events and the interaction timing of the icub robot were predefined. We put markers for the concrete time stamps in the analysis charts provided in later sections.

## 3. Analysis Tools and Their Application

During the postprocessing of our video recordings, we removed two participants from the *no-robot* condition (13 persons), one participant from the *non social* condition (14 persons), and one participant from the *social* condition (14 persons) due to severe image corruption.

### 3.1. The Arousal-Valence Dimension

The measure of affective states in terms of arousal and valence extracted from facial expressions has become an important tool in descriptions of facial expressions. The term valence is usually described as the degree of pleasantness, while arousal signifies the amplitude. Their values vary continuously between [−1;1] signifying e.g., “excitement” (arousal/valence both positive) or “boredom” (arousal/valence both negative). The neutral state is represented by (0, 0).

For the extraction of arousal and valence, we employed the “FaceChannel” deep neural network architecture introduced by Barros et al. (2020). In the FaceChannel network, each image or video frame is passed through a cascade of convolutional and pooling layers including an inhibition mechanism before passing to the classification stage. Although the architecture resembles the classic VGG16 network (Simonyan and Zisserman, 2014), the FaceChannel needs fewer numbers of network parameters to train to achieve similar performance. Therefore, we can extract the arousal-valence profile from the participants in real time.

### 3.2. Gaze Behavior

In addition to arousal and valence, we are also interested in the gaze behavior of participants to detect possible distractions induced by the robots' presence. The OpenFace software was developed by Baltrusaitis et al. (2018) which offers a rich set of pre-trained models for the detection of the face (respectively facial landmarks), eyes, and the calibration to obtain the gaze direction and head movements. Therefore, the output provides a large feature set from which we selected the eye gaze behavior in the {*x, y*} direction, averaged over the two eyes (Baltruvsaitis et al., 2015; Wood et al., 2015). To obtain the rate of change in the {*x, y*} direction, we first converted the output from radians (*rad*) to degrees (°) and then calculated the frame-wise differences.

### 3.3. Facial Action Units

The “Facial Action Coding System” was first introduced by Paul Ekman (Ekman, 1970) and represents facial muscle activity involved in emotion expressions, the individual action units (AU). Table 1 summarizes the AU labels for specific facial muscle parts and Table 2 shows the involvement of particular AUs when emotions are expressed^3^.

**Table 1:** Action unit code for specific face muscle activity.

AU1	Inner brow raiser
AU2	Outer brow raiser
AU4	Brow lowerer
AU5	Upper lid raiser
AU6	Cheek raiser
AU7	Lid tightener
AU9	Nose wrinkler
AU10	Upper lip raiser
AU12	Lip corner puller
AU14	Dimpler
AU15	Lip corner depressor
AU17	Chin raiser
AU20	Lip stretched
AU23	Lip tightener
AU25	Lips part
AU26	Jaw drop
AU28	Lip suck
AU45	Blink

**Table 2:** Action units involved to express basic emotions.

Emotion	Action units active
Anger	AU 2, 4, 7, 9, 10, 20, 26
Disgust	AU 2, 4, 7, 9, 15, 17
Fear	AU 1, 2, 4, 5, 15, 20, 26
Happiness	AU 1, 6, 12, 14
Sadness	AU 1, 4, 15, 23
Surprise	AU 1, 2, 5, 15 16, 20, 26

Similarly for the gaze, we used the OpenFace software for the extraction of the AUs for each participant which allows us also to correlate the activity of affective-specific face muscles with the output of arousal-valence from the FaceChannel. For instance, we can analyze whether positive arousal-valence like “excited” correlates with the activity of AUs involved in expressing “happiness.” This way, we can verify the robustness of both tools and ensure proper interpretation of our results. In total, 17 AUs are extracted with their intensity levels in a continuous range [0;5]. All action units are also available as binary features (presence/not presence) but the intensity level of an AU does not necessarily align with its presence due to different datasets used for training. Therefore, we skip using the binary features except for AU28 (“lip suck”) which is only available in this form. Finally, we also extracted the so-called “confidence” in [0;1], which denotes the success of face tracking. A reliable tracking result is achieved for confidence ≥0.7^4^.

## 4. Results of Affective State Indicators

In this section, we present an evaluation of significant facial features obtained from the tools described. Here, we focus on the behavior over the whole experiment, while in the next section, we concentrate on the temporal analysis and event-based correlations.

### 4.1. Eye Gaze Patterns

We computed the eye gaze behavior as the rate of change of gaze angles during the experiment. In general, gaze values close to 0 denote no change in the gaze. A change on the *x*-axis from positive to negative assigns a gaze movement from left to right; and if a person looks from up to down yields a change of the *y*-axis from negative to positive (Baltruvsaitis et al., 2015). However, we do not use this information as we are not interested in the concrete gaze pattern to evaluate eye trajectories. Our aim is to detect possible sudden changes of the gaze during task performance and especially in cases when the robot displays behaviors as in the *non social* and *social* conditions.

**Figure 2:**
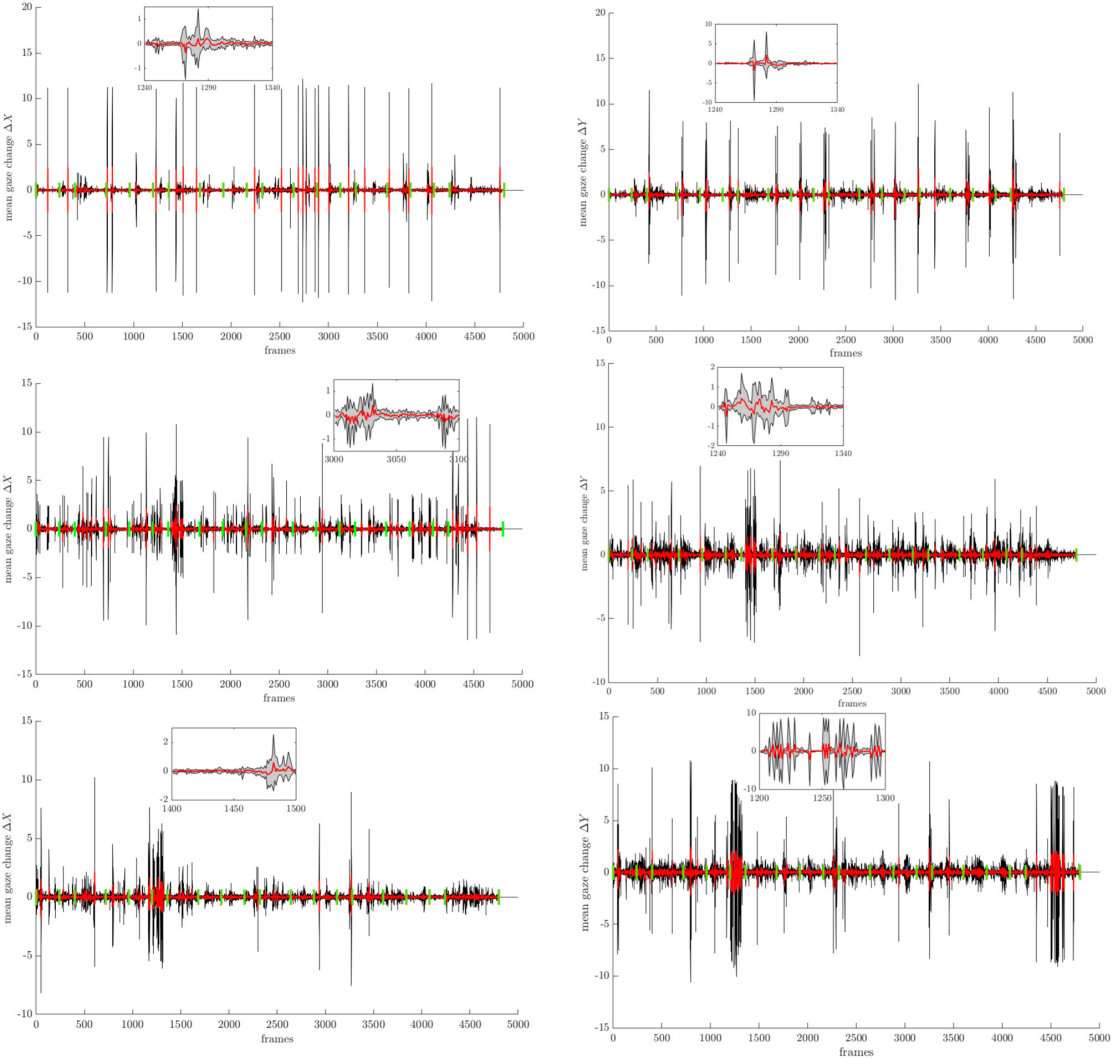
Rate of change of the gaze angles in ° in {*x*} and {*y*} direction (pitch; yaw), averaged over all participants for the three conditions. The inset shows the filtered signal with the mean curve (red) and the standard deviation. The green vertical bars denote the task onsets (frame level).

Figure 2 demonstrates the average rate of changes in the *x, y* (pitch; yaw) direction denoted by Δ*X* (first column) and Δ*Y* (second column), in degrees, over all participants for all three conditions. The green vertical bars denote the time onset for a new task from the MATB protocol. For a better visualization, we provide exemplary insets which show the filtered mean signal and the SD. We observe only small fluctuations of the changes in the gaze behavior along the x-axis for the *no-robot* and *social* condition mostly around the task onset, i.e., when a new task starts. The participants in the *non-social* condition elicit more eye movements in general and specifically at the start and end of the experiment. Along the y-axis, we see an increase of gaze changes from the *no-robot* to *social* condition, the latter showing a peak at the onsets of task 5 and high activity at the end of the experiment. However, overall the participants elicit only small fluctuations in contrast to the *non-social* condition, where the gaze changes occur more continuously. Overall, it should be noted that the gaze changes remain in a relatively small range of ±10° and mostly after task onsets.

### 4.2. Analysis of the Arousal-Valence Dimension

We first present the distribution of arousal and valence for the three conditions over the whole experiment. The evaluation follows the characteristics of the arousal-valence dimension as described in Section 3.1.

#### 4.2.1. No-Robot Condition

**Figure 3:**
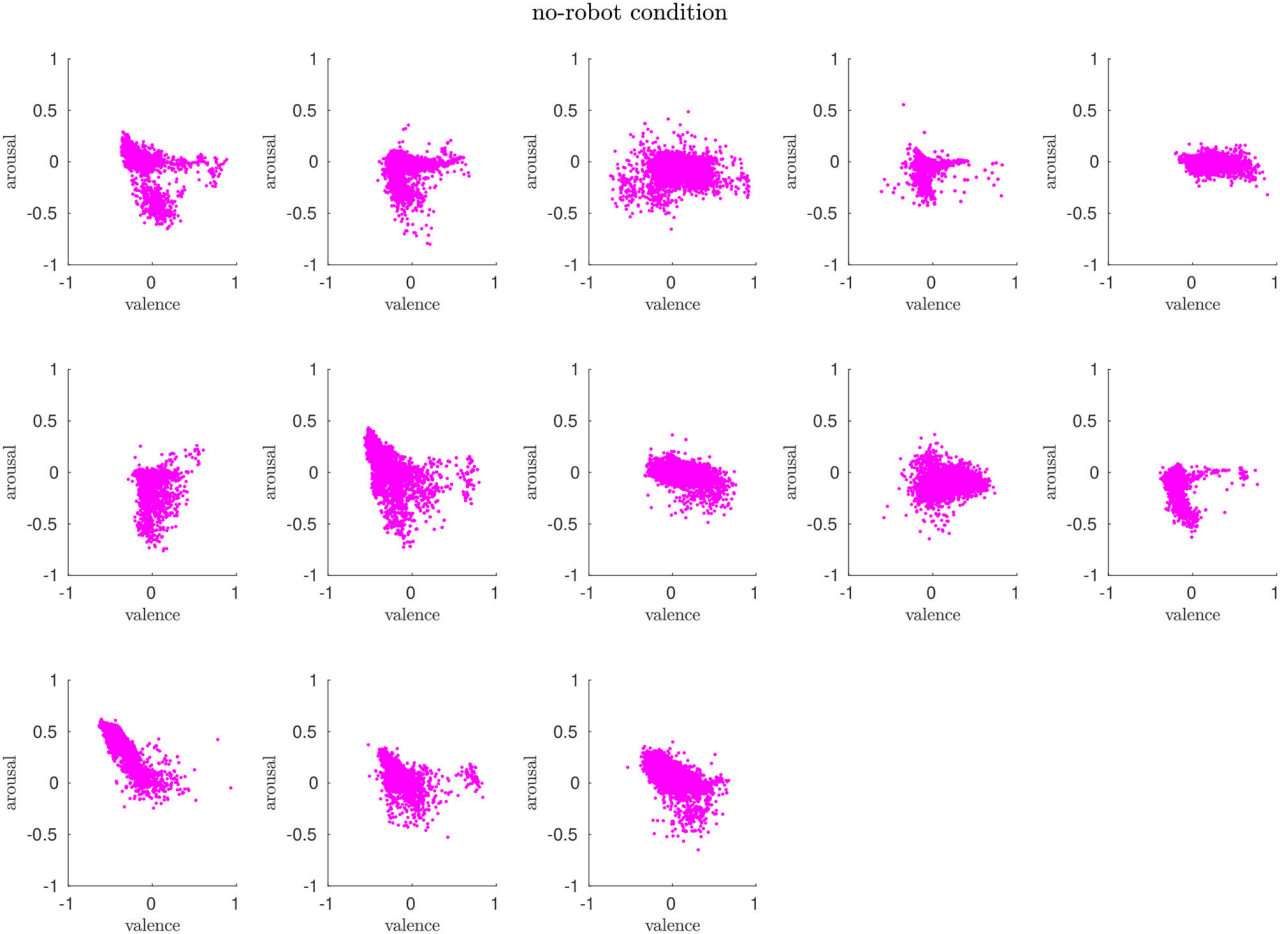
In the *no-robot* condition, the participants' affective states are mostly clustered around the neutral state and evolve around the horizontal axis (valence).

Overall, the arousal and valence for around half of the participants in this condition are distributed mostly around the neutral state (0, 0) with diffusion toward negative arousal (vertical axis). For some participants, we observe a rather horizontal scatter profile, which means that their valence values change between positive and negative. We detect a clear trend toward negative arousal for three participants. The results show that humans in this condition do not elicit strong negative or positive affective states but rather routinely perform the task. Figure 3 shows the scatter plots of all participants.

#### 4.2.2. Non-social Condition

**Figure 4:**
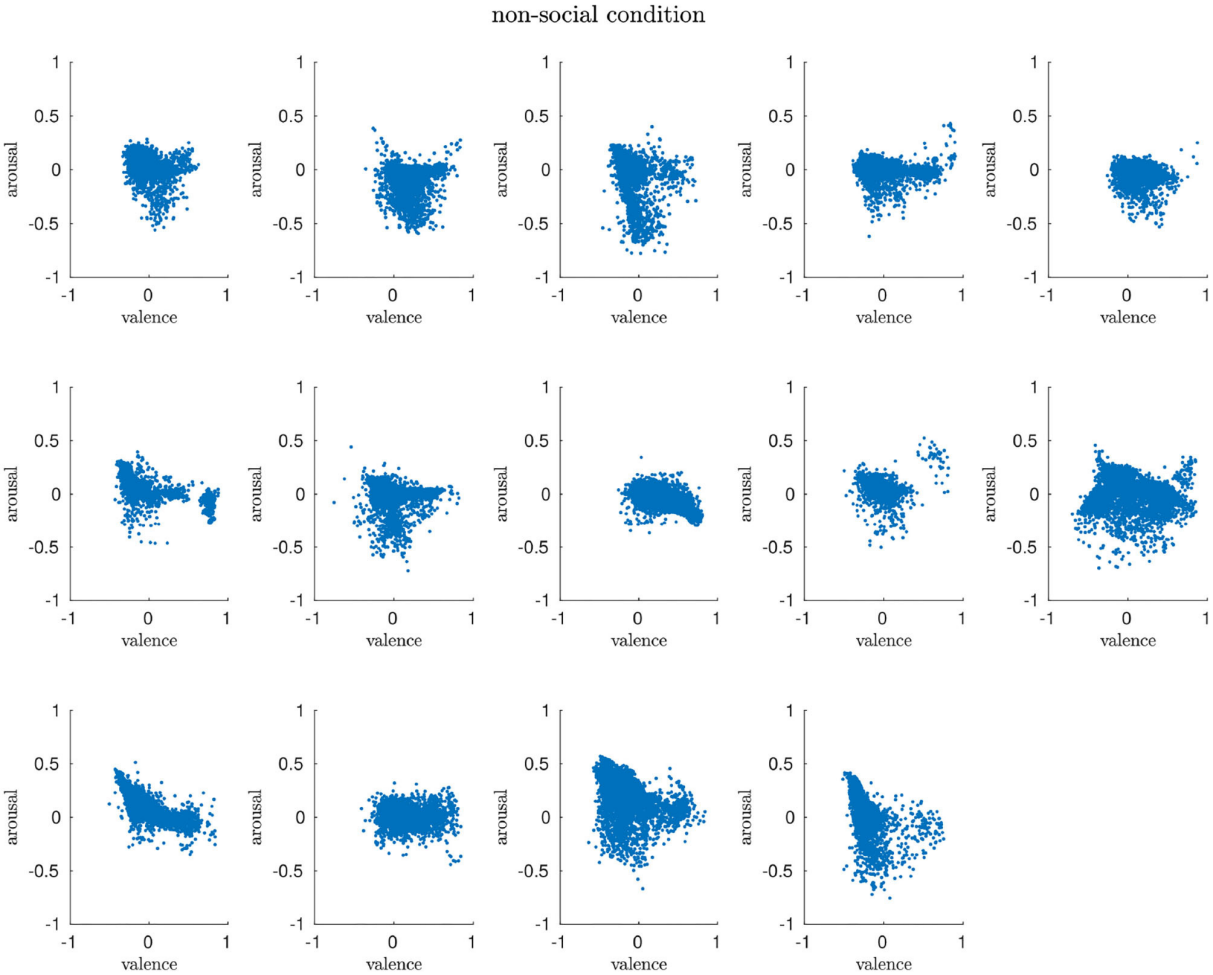
The arousal and valence patterns in the *non-social* condition are very diverse, covering a huge space of the arousal-valence dimension.

We can find similar arousal and valence distributions among the participants for the *non-social* condition, especially demonstrated by the vertical trends, i.e., low arousal. However, the scatter profile show more variance, i.e., the clusters are bigger than for the *no-robot* condition and cover both negatively associated parts of the arousal-valence scheme as well as areas that are identified to code for positive affects. Our interpretation of this evaluation is that the presence of the robot does have an influence on the participants but that the rather mechanistic behavior triggers both negative and positive affective responses in humans. We will elaborate on this aspect in more detail in a later section. Figure 4 shows the scatter plots of all participants.

#### 4.2.3. Social Condition

**Figure 5:**
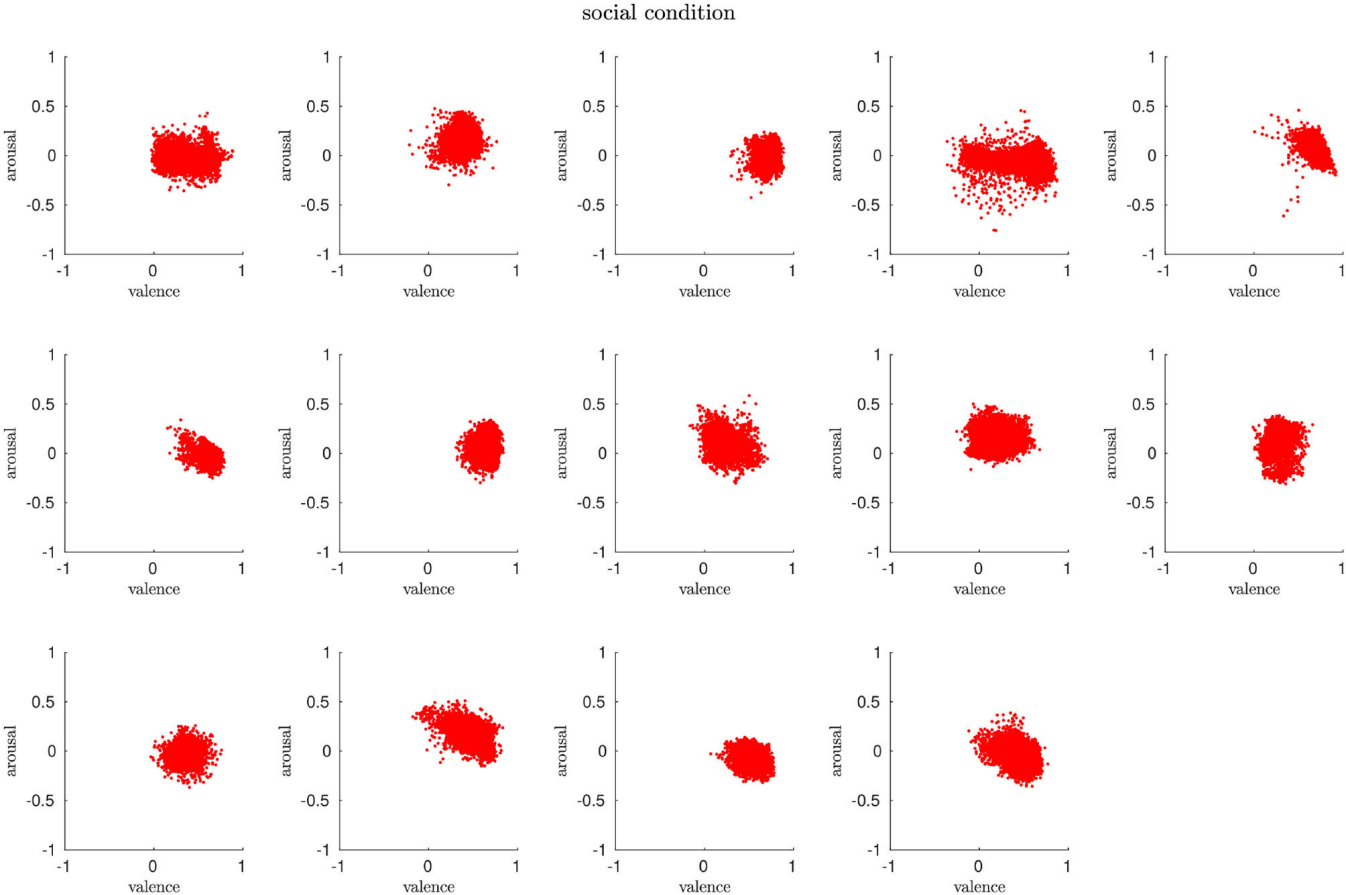
Distribution of arousal and valence over all participants in the *social* condition. The affective states are mostly clustered toward positive values and are more compact compared to the *non-social* condition.

The distributions for arousal and valence in this condition show predominantly compact clusters, which means that the majority of participants are in a calm state and, based on the positive trends on the valence axis, display a higher level of pleasantness than in the previous two conditions. Due to the low variance of clusters on the arousal-valence axes in contrast to the mixed profile for the *non social* condition, we are encouraged to identify the social behavior as the specific impact on humans during task performance. This is further supported by the absence of signs of monotonicity, sleepiness, or even boredom as shown for the *no-robot* condition (significant distribution along the vertical axis toward low arousal and low valence) nor trends toward high negative arousal as detected for the *non-social* condition. Figure 5 shows the scatter plots of all participants.

#### 4.2.4. Individual and Group Analysis

We are interested in the affective states of the participants both within each condition and between the conditions. We first compute the statistical significance for each group using the kruskalwallis function from Matlab (MathWorks). For all three conditions and a significance level of α = 0.01, our results do not support the null hypothesis, i.e., the arousal and valence profiles differ significantly between the participants and per condition (*p* = 0). We use the paired sample *t*-test ttest2 (α = 0.01) to compare the arousal and valence distribution for the three conditions. Our results reveal statistically significant differences (*p* = 0) between the conditions, which supports that the presence of a robot and the interaction style impact the affective states.

### 4.3. Analysis of Action Units

While arousal and valence are continuous values characterizing a certain affective state, using action units (AU) corresponds to discrete emotion classification using the Facial Action Coding System (FACS). FACS has the advantage that particular facial muscles can be identified which are active during a certain emotional facial expression. For instance, the eyebrows contract when expressing “anger” or the lip corners are pulled up when we are happy and smile. Although the FACS is based on Ekman's notion of cultural-independent basic emotions (Ekman, 1970), the composition of different muscles to express facial emotions as well as their intensity remains individual and can vary across cultures. For this study, we extract 17 action units (AU) from the Openface software with their corresponding intensity values in the continuous range [0;5] (Baltruvsaitis et al., 2015). Additionally, we consider AU28 coding for “lip suck,” which is only available in binary format (presence/absence) (Baltrusaitis et al., 2018). We follow the abbreviation scheme as shown in Table 1. First, we created heat maps for the action units over all participants in each condition to display their overall occurrences during the experiment. First, we binarized the intensity-level AU data as the AU detection module of the OpenFace software has been trained on different datasets, thus the outputs may differ. We then simply summed up the occurrences of each action unit over all frames and divided the results by the maximum frame length (4,801 = 300 s*16 fps) to normalize the value range into (0;1).

#### 4.3.1. No-Robot Condition

**Figure 6:**
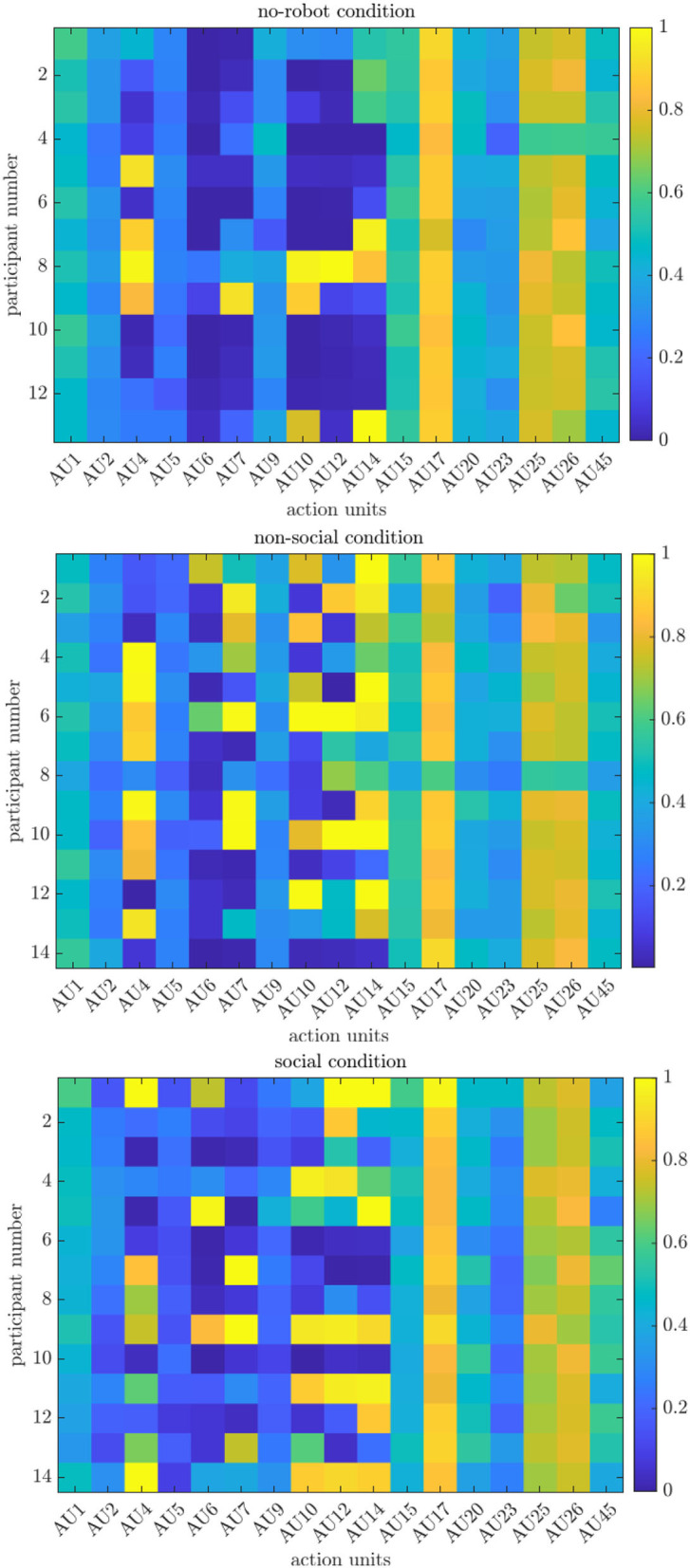
Normalized heat maps of action unit intensity levels over all participants for all experimental conditions.

Figure 6A shows the normalized number of occurrences for all action units (AU, x-axis), which follow the FACS coding from Table 1, over the 13 participants (y-axis). The value 1 signifies that an AU always appears. We observe the lowest participation for AU2 (outer brow raiser), AU5 (upper lid raiser), AU6 (cheek raiser), AU7 (lid tightener), AU9 (nose wrinkler), and AU23 (lip tightener). Except for the latter, these action units belong to muscles in the eye region which are an integral part of the composition of emotional expressions as summarized in Table 2. Similarly, AU4 (brow lowerer), AU10 (upper lip raiser), AU12 (lip corner puller), and AU14 (dimpler) are mostly absent (with some exceptions for a few participants). These action units are involved in the emotion categories “anger” and “happiness”, where for the latter the activation of AU12 and AU14 is exclusively relevant. The absence of these action units let us rule out the “happiness” state of participants for this condition. Analyzing the active action units, we observe a medium activation (≈0.4 − 0.6) of AU1 (inner brow raiser), AU15 (lip corner puller), AU20 (lip stretched), and AU45 (blink). The former three action units are involved in the expression of “fear,” “sad,” and “surprise.” Given that the task is not anxiety-inducing nor do the participants have to fear a negative reward, we tend to interpret this result in favor of the “sad” or “surprise” category. This is further underpinned by the presence of AU4 (brow lowerer) for the participants 5, 7, 8, and 9, which refers to the “sad” emotion and strong involvement of AU26 (jaw drop) in expressing “surprise.” We understand the rather average participation for the blink behavior expressed in AU45 as a common pattern in experiments. Counter-examples would be the lack of blinking, i.e., low intensity level which can occur in phases of high concentration or high blinking frequency, i.e., high intensity level, when humans are exposed to stress (Haak et al., 2009). Finally, we see high occurrences for the action units AU17, AU25, and AU26. Surprisingly, the “chin raiser” activation, AU17, engages in the expression of “disgust.” However, this emotion class comprises other facial muscles which we find mostly absent in our analysis. Therefore, we would object to the interpretation of this class. In addition, AU25 (lip part) is not represented in any of the emotion categories. After a *post-hoc* video analysis, we conclude that AU17 and AU25 are rather involved in the expression of “surprise,” although these action units do not appear in the emotion listed in Table 2. The major activation of AU26 “jaw drop” is an essential unit for the “sad” and the “surprise” category and its occurrence underlines the cognitive load induced by performing the MATB task. To quantify also the AU detection, we analyze their intensity levels which are in the range [0;5]. We observe that for all action units, their activity dampens after the experiment starts and rises again after experiment termination. During the time course of the actual experiment, most of the action units display only temporal activation or spike-like patterns, with the exception of AU17, AU25, and AU26. However, all action units' activation falls within a small range of intensity up to only ~2.

In general, our results for the *no-robot* condition adjust with our analysis of the arousal-valence distribution: the participants are predominantly in a neutral state demonstrated by rather low activations of the action units, which is shown in a vertical spread of arousal-valence in the corresponding scatter chart **3**. Some of the participants show clear activation of facial muscles involved in “surprise” or sad, which aligns with a trend to the left of the arousal-valence values, respectively, downward shift to low arousal and negative valence. Finally, we detect no “happiness” emotion, which is also underpinned by the absence of trends toward the positive division of the arousal-valence chart.

#### 4.3.2. Non-social Condition

Overall, both the participation of action units as well as the distribution of their intensity is more diverse than for the *no-robot* condition. Similar properties shared between the two conditions are the low activation of action units related to the eye region, namely AU2 (outer brow raiser), AU5 (upper lid raiser), AU6 (cheek raiser), and AU9 (nose wrinkler). Similarly, we observe medium intensity for AU15 (lip corner depressor), AU20 (lip stretched), and AU45 (blink) as well as the highest activation over all participants for AU17 (chin raiser), AU25 (lip part), and AU26 (jaw drop). However, in contrast to the *no-robot* condition, the action units AU1 (inner brow raiser), AU4 (brow lowerer), AU7 (lid tightener), and AU10 (upper lip raiser) display higher intensity levels, which are represented mainly by the emotions “anger” and “sad.” However, it should be noted that individual action units appear in multiple emotion classes. Especially AU1 (inner brow raiser), AU15 (lip corner depressor), and AU20 (lip stretch) are involved in four, respectively, three out of the six categories. Hence, we also include the category “surprise” into our interpretation, while ruling out “disgust” (absence of AU9) and “fear,” the latter based on the same argument as explained above. Another critical difference observed in this condition compared to the *no-robot* condition is the increased participation of AU12 (lip corner puller) and AU14 (dimpler), both expressing “happy.” In addition to the AU detection, we also analyze the concrete intensity-levels per AU. We observe that similarly to the *no-robot* condition, the intensities spike around or evolve continuously over the experiment time not more than a value of 2.

#### 4.3.3. Social Condition

In the *social condition*, we observe that participants display low expressions of AU2 (outer brow raiser), AU5 (upper lid raiser), AU9 (nose wrinkler), and AU23 (lip tightener). Interestingly, the detection of action unit AU4 (“brow lowerer”) is separated into two groups, either with low or high participation. Similar to the *non-social* condition, we observe also increased values for AU1 (inner brow raiser) and AU10 (upper lid raiser), as well as moderate levels for AU15 (lip corner depressor), AU20 (lip stretched), and AU45 (blink) comparable to both conditions. Furthermore, we observe higher expressions for action units AU6 (cheek raiser), AU12 (lip corner puller), and AU14 (dimpler), which, in conjunction, represent the “happy” emotion category. Strikingly, AU17 (“chin raiser”), AU25 (“lip part), and AU26 (“jaw drop”) are also the predominant action unit in this condition. While the major occurrence of AU26 can be explained by its involvement in the expression of “anger” and “surprise,” we do not find evidence of “disgusted” participants although AU17 is the unique action unit for this class. Also, we cannot answer why AU25 is central but absent in the emotion list shown in Table 2. From our analysis, we would, therefore, conclude that both action units are either part of the “surprise” class or involuntary facial expressions due to the cognitive load induced in the MATB task. The quantification of the AU participation by their intensity levels shows increased activation of AU1, AU2, AU6, AU12, and AU25, which are involved mainly in the expression of “surprise” and “happy.” However, the intensity of the expression of the other action units is comparable to the other two conditions.

Overall, the participation of action units is more pronounced in the *social* condition compared to the *no-robot* condition, yet less diverse than in the *non-social* condition. Again, this result aligns well with our qualitative analysis of the arousal-valence distributions, which show more clusters around the 1st quadrant of the dimension chart that represents positive affective states. While Figure 6 also demonstrates the participation of facial muscles involved in “anger” and “sad,” it is important to reveal the time context, i.e., whether the action units appear together. We will clarify the temporal progress of the affective states and the occurrences of action units in the next section.

## 5. Temporal Correlations Between Task Onset and Affective States

In this section, we present a temporal analysis of the time context for both arousal-valence and action units. Specifically, we study whether significant changes in the intensity of the affective states, respectively, facial expressions are displayed concurrently to the task onset (events) or whether we can find evidence of the robots' impact on changing the humans' behavior. Due to the image-wise processing of the employed software, the time component in our analysis is on the frame-level. In our graphics, the label “start” corresponds to the experiment starting at frame 0; after 15 s, the first event begins which corresponds to frame number 240 (15 s * 16 fps frame rate), and the second event starts at 25 s which corresponds to frame number 400 and so on, after the 5 min per experiments have been reached (marked with “end” in the figures).

**Figure 7:**
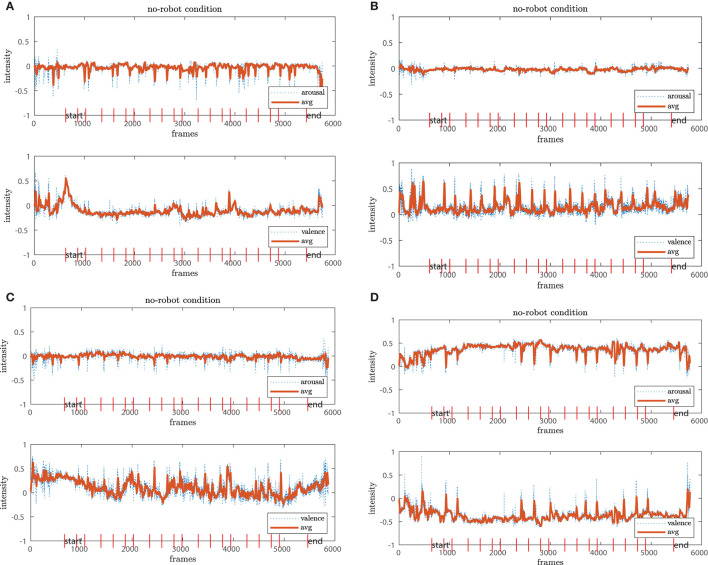
Representative temporal profiles of the arousal-valence progress in the *no-robot* condition. While the arousal state remains stable **(A–D**, upper plot**)**, the valence dimension fluctuates for some of the participants, mostly in agreement with task onsets **(B–D)**.

**Figure 8:**
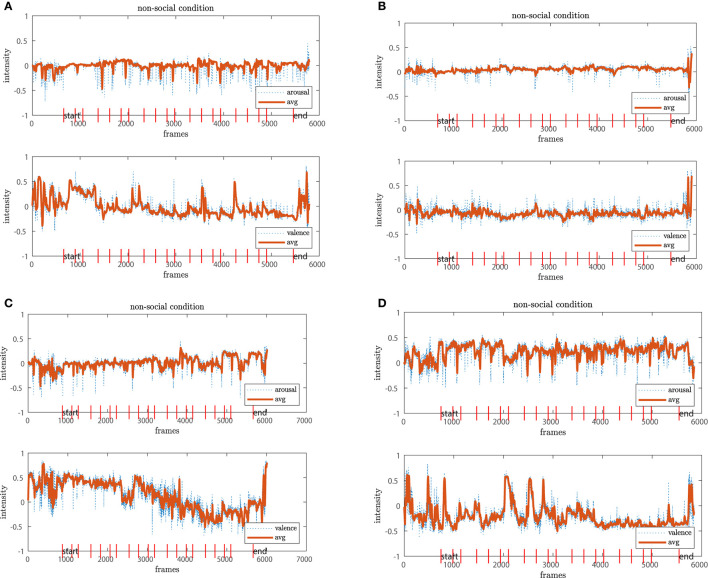
The *non-social* condition reveals higher fluctuations in both dimensions. While in chart **(A)** both arousal-valence seem stable, there are higher amplitudes for **(B–D)**. In **(C)**, we observe even an downwards trend in the valence dimension over the time course of the experiment.

**Figure 9:**
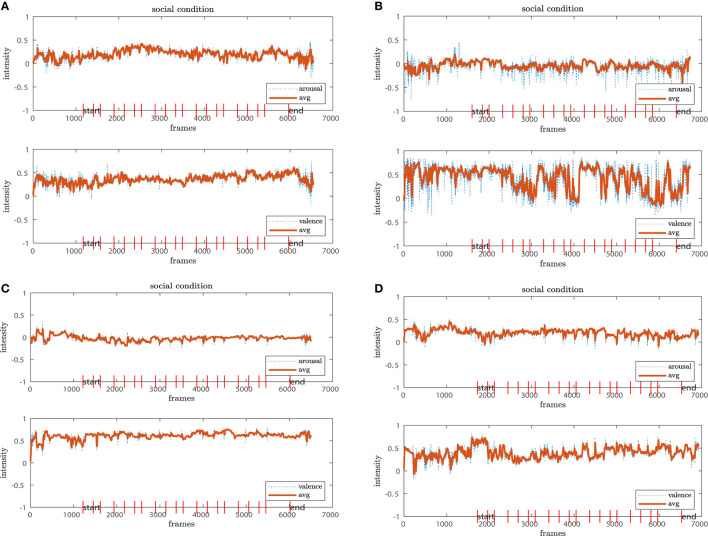
The participants in the *social* condition show stable arousal-valence patterns with positive trends **(A–D)**. The result aligns with the positive clusters shown in the scatter plots.

For the analysis of arousal-valence, we first filtered both individually with a moving average of window size 16 to account for the 16 fps recordings. From this filter step, we select four exemplary profiles from participants for each of the conditions whose evaluations are representative of their group, shown in Figures 7, 8, 9A–D. The red vertical bars represent the task onsets.

We observe that the arousal dimension has a low activity for the *no-robot* and *social* conditions while participants in the *non-social* condition display a more diverse pattern. In connection with the valence dimension, we see that participants in the *no-robot* condition tend to be more neutral or show higher activation at the onsets of events. In contrast, participants in the *social* condition show more stable positive valence with one exception where the participants' valence fluctuates strongly between positive and negative. The results from the *non-social* condition vary in intensity amplitude from the very low activity for both arousal and valence (neutral or calm state) to high intensities which is usually interpreted as an “angry” or “annoyed” state. The latter is less pronounced in the other two conditions. Additionally, we observe a negative trend in Figure 8C from high to low valence with a relatively stable arousal pattern. A similar pattern is detected for the 4th participant, where high peaks occur at the early experimental stages and reduce continuously over the time course. We interpret this result that either the participants adapted to the experiment and the environment feeling more confident or comfortable or become calm or even bored.

In general, the different coverage of affective states in the arousal-valence dimension underpin our results from Section 4.2; similarly, we observe a narrow distribution around the neutral to calm or bored affective states in the *no-robot* condition compared to the *social* condition, which comprises mostly positively connoted affective states over the time course of the experiment. The diversity of affective state expressions in the *non-social* condition is also represented in the temporal process. However, the graphs also demonstrate that low or high activity profiles occur in all three conditions, independent of the robots' presence or behavior. These observations need to be taken into account to disentangle the real role of the robot in the experiment.

As the figures only allow qualitative analysis of the temporal behavior, we also implemented a simple change point detection scheme. First, we compute the difference between two consecutive frames which is high when either a peak or a sudden drop appears in the signal, i.e., the intensity level of action units. Then, we extract the frame index and its associated value from such a change point. We sort the maximum values in descending order and extract only the first 20 indices as we have 20 events (start/end included). We do this for all participants over all conditions so that we can compare those indices with the event onsets. Specifically, we can check whether or not the most significant changes in AU activation align with the task onsets, respectively, and how much the detected changes diverge. A high divergence from the predefined experiment timing may allow us to find links to the robotic behavior for the *non-social* and *social* behavior. Due to the combinatorial nature of the evaluation (*#conditions* × *#participants* × *#AU*), we select representative samples from the temporal analysis and concentrate on the evaluation of the most active action units as described in the previous section. The criterion for the representative selection is to demonstrate the whole spectrum of participant responses per condition. This way, we want to avoid the selection bias of our analysis (e.g., showing only selective responses that show a specific affective state).

### 5.1. No-Robot Condition

**Figure 10:**
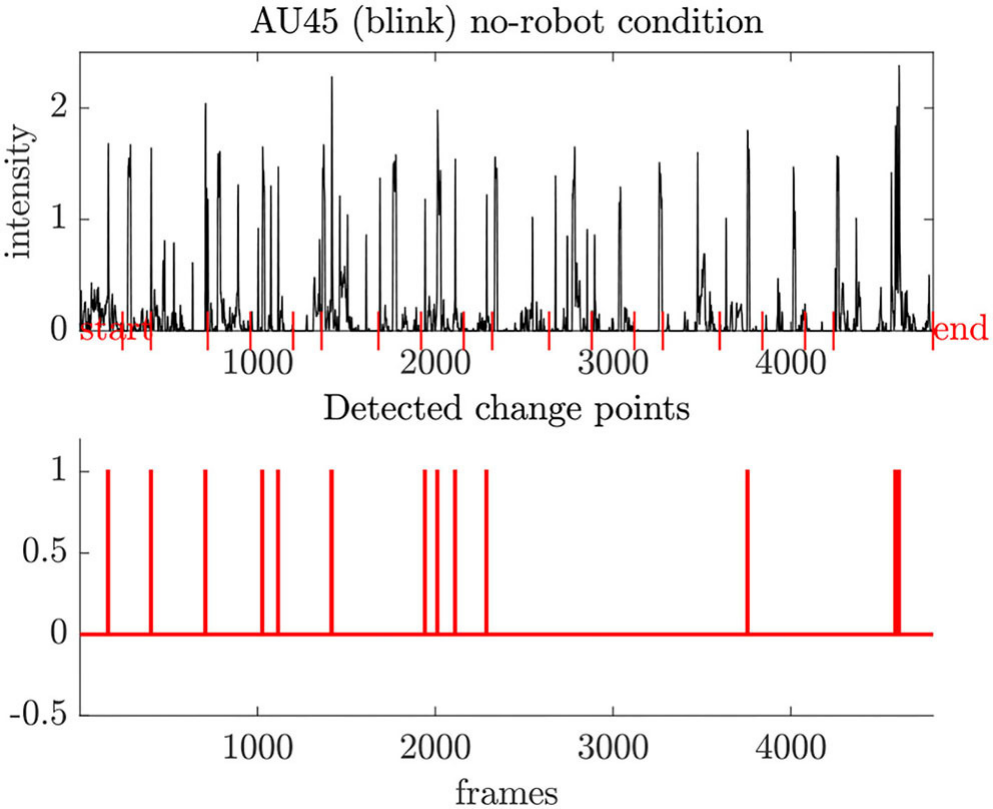
Typical blink pattern (AU45) for participants during the experiment. Our change point detection scheme reveals most activity related to the task onset.

Most facial expressions shown by the participants emerge from the lip part (AU15, AU23, AU25) and often relate to the task onsets during the experiment, i.e., their activity is characterized by a spiky pattern. Using our point detection scheme, Figure 10 shows that the blinking behavior (AU45) is in agreement with the task timings, and we do not observe hints of stress (high blink frequency). However, the appearance of some blink gaps may indicate that the participants concentrate on the task performance.

To see any agreement between the results obtained for arousal-valence, which was revealed to be mostly neutral in the affective dimension, we also compare the concurrent activation of the emotion class “happiness” (AU1+AU6+AU12+AU14) and the class “anger” (AU2+AU4+AU7+AU9+AU10+AU20+AU26). We analyzed the AU synchrony both qualitatively by plotting the AU intensity charts in relation to the task onsets. Also, we compute the cross-correlation between the different AU signals as a function of some time lag *k*. In brief, the cross-correlation measures the pairwise similarity between signals and is highest, i.e., 1, if both signals are equal. For “happiness,” we observe the following: the overall intensity levels of the corresponding AUs are rather low. Almost no participants show concurrently activated action units, leaving sparse intensity patterns with mostly AU1 (inner brow raiser) activated. AU6 (cheek raiser) shows the least participation. In the case of co-activations, they mostly occurred right after a task onset, i.e., the presumable expression of “happiness” relates to a successful performance of the human. The cross-correlation at lag *k* = task onset underpins the observations; the correlation values comparing the action units range maximum up to ≈0.2. The frequency of AU14 (dimpler) for some participants occurred either related to an event or continuously over the whole experiment. Although these patterns are exceptions in the facial expressions compared to the majority of participants, we find this result in agreement with some positive expressions revealed by the arousal-valence evaluation. Therefore, we interpret the occurrence of AU14 as an expression of either an involuntary externalization of the thinking process, a reaction to a possible mistake made by the human, or, in the case of positive response, the personality style of the person. We can detect no signs for concurrent action unit activations involved in expressing “anger” but discover most activations for AU2 (outer brow raiser), AU20 (lip stretched), and AU26 (jaw drop) which are comprised in the “surprise” class. This observation is further underpinned by the absence of AU10 (upper lid raiser), a unique action unit in the “anger” class.

### 5.2. Non-social Condition

The diversity in the different amplitudes revealed by arousal-valence is also mirrored in the evaluation of the “happiness” emotion. Again, we first analyze the AU signal co-occurrences which compose a “happy” emotion and observe both high synchrony of the action units as well as very sparse AU activations which, moreover, do not align. The cross-correlation coefficients which range from low to high values related to task onset and beyond underpin the impressions. The synchrony analysis of AU expression for the “anger” emotion shows that action units are co-activated either around the task onset (mostly delayed over some frames) or occur also between the tasks. Also, we detect rather sparse patterns, which means that some of the participants show no signs of “anger.” In some cases, there are no concurrent intensity spikes from the action units but the main activation of AU7 (lid tightener) and/or AU10 (upper lip raiser). However, the two action units only appear for the “anger” class, thus we might observe cases where the software fails to detect reliably well all AU activations. Finally, we see an inverse relationship between AU7 and AU10 with AU26 (jaw drop). We can this result by either mentioned failure of the OpenFace software due to face position issues or those participants rather elicit “surprise” reactions than being angry. The latter aspect would align with our observation from the heatmap demonstrating the number of activated action units over the experiments.

### 5.3. Social Condition

**Figure 11:**
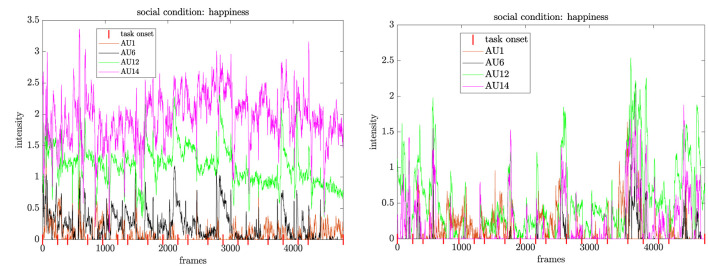
Concurrent activation of action units representing the “happiness” class. While most of the co-activation appears at task onset, the activation decays slowly in between two tasks. This pattern might display a positive relation between the human and the socially-behaving robot.

For change points in AU17 and AU26, we see most changes at the beginning of the experiment, which can be explained by the surprise effect of both the robot behavior and the adaption to the first tasks. We observe normal blinking behavior expressed in the activation of AU45, i.e., most changes in the blink behavior align with the event onsets. Our analysis of arousal-valence revealed a stronger trend toward positive affective states shown by the compact scatter plot clusters. Additionally, the temporal charts from selected participants reveal that the positive attitude endures throughout the time course of the experiment. For the emotion expression by action units, we opt for an agreement between them and our results obtained for arousal-valence. Therefore, we analyze when the action units representing “happiness” occur concurrently and whether this is synchronous across the time line of the experiment. In fact, we find that the action units co-occur for most of the participants as shown in Figure 11. In most cases, the participants elicit a “happy” expression short after an event and stay within this expression beyond the onset. As the robot behaves socially including talking to the participants, which induces some temporal delay, we conclude that the small decline of the intensity curves is linked to a positive experience for the human with the robot. For four participants, we identified a sparse intensity pattern with most activation from AU1 (inner brow raise) and AU6 (cheek raiser) after task onset. Interestingly, however, is that in between the task the participants also show some activation of AU12+AU14 (lip corner puller + dimpler), indicating that there might be some positive impact from the robot. In the analysis of the “anger” class, we detect mostly activations for AU2 (outer brow raiser), AU20 (lip stretched), and AU26 (jaw drop), and, over all participants, least occurrences of AU7 (lid tightener) and AU10 (upper lid raiser). Therefore, the participants rather show expressions related to “surprise” than “anger.” Overall, we could not see concurrent activation of all the seven action units. The patterns for facial expressions involved in “anger” like co-occurrences of AU4 (brow lowerer), AU7 (lid tightener), AU9 (nose wrinkler), and AU10 (upper lip raiser) for some of the participants are spike-like around the task onset and decrease quickly. As the humans seem to recover from this emotion and do not seem to carry it over to the behavior of the social robot, we can conclude that either the participants solely concentrate on the task or that the robot has a positive impact on their emotional response. The latter aspect can be confirmed by the temporal profiles of the arousal dimension, where we observe only small arousal levels in some of the participants. Correspondingly, those participants might find the social presence of the robot pleasant but do not necessarily express it.

## 6. Interpretation of Results

Our analysis revealed interesting behaviors that we are going to discuss in light of the interplay between the experimental conditions and the actual cognitive task.

### 6.1. Eye Gaze Pattern

We interpret our results obtained from the eye gaze analysis from two sides: first, we can expect a rather stable gaze pattern across participants and conditions as we constrained the gaze radius in the tracking task. Furthermore, the role of the robot was to accompany the participant through the experiment, while the participant concentrated on the task itself. This design differs from HRI experiments with mutual interaction involving, e.g., dialogues where participants have a proactive role toward the robot (Kompatsiari et al., 2017; Pereira et al., 2020). On the other hand, the icub robot shows some nonverbal and verbal behavior in the *non social* and *social* conditions. Participants less experienced or exposed to robots may be affected by these behaviors. As a result, one might consider a stronger trend toward changes from left to right (the direction from the participants' view) during the whole experiment to confirm or observe what the robot is actually doing. However, our results do not confirm this thought, so we conclude that the robots' behaviors during both conditions do not negatively impact the human performing a cognitively demanding task. This result is particularly interesting for the *social* condition as it gives us hints to believe that the participants attributed the robot to be a companion-like partner.

### 6.2. Arousal-Valence and Action Units

For the *no-robot* condition, our analysis revealed arousal patterns mostly along the horizontal axis (valence), which means that the participants mostly change their level of perceived pleasantness. However, for some participants, we also observe changes in the arousal level, mostly in the negative direction. In connection with the temporal arousal-valence profile, which shows regular peaks at the task onset, we conclude that the participants focused strongly on the task but either do not elicit strong affective responses or show some level of annoyance during task performance. Interestingly, the temporal profile also how that the affective behavior attenuates quickly. This behavior is expected as the participants cannot share their affective experience neither with another human nor with the robot. However, especially the scatter plots demonstrate that even in a rather short experiment (5 min) and without interactions, humans tend to differ in the amplitudes of their affective emotional expression levels. The analysis of the action units let us conclude that the activations of action units 17 and 25 (chin raiser, lip part) are rather involved in the expression of “surprise,” although AU17 has been identified merely in the “disgust” class. This is confirmed by high activations of AU26 (jaw drop) among all participants.

In contrast to the baseline condition, the arousal-valence distributions shown in Figure 4 show higher diffusion of affective values, which make up a bigger set of affective states than for the baseline condition. Overall, there are trends toward positive and negative arousal (vertical axis) for the majority of participants. Similarly, the temporal profiles vary significantly among the participants with the most activity at the task onset. Again, the amplitudes in the expression levels differ but conversely to the *no-robot* condition; here, only a few participants are rather calm. Our results may indicate that the humans in this experiment are confused by a humanoid looking robot, awakening some expectations like interactions or following the social etiquette, which the robot due to its condition fails to deliver. The discrepancy in appearance and behavior in conjunction with a cognitively demanding task may elicit stronger affective responses than in absence of a robot. The participants in the *non-social* condition exhibit more facial expressions which reveal a diverse emotion pattern extended by the presence of muscle activity involved in the expression of happiness. The observation for the action units aligns with the results obtained from the arousal-valence analysis and supports our previous interpretation. Given that the robot has anthropomorphic features but shows no social behavior, it can trigger different responses in the humans based on a possible mismatch between the appearance and behavior of the robot, thus the people may tend to show “anger” or “sadness” as shown by the activation of corresponding action units. On the other hand, participants attributing the mere presence of the robot as a companion may feel encouraged to share also positive feelings, e.g., in case, the task has been performed correctly, in contrast to participants in the *no-robot* condition. We also observed higher values of action units around the task onset and between two tasks, i.e., the action units analysis confirms our interpretation that there is a separation between the participants into a group that concentrates on the tasks (similar to the *no-robot* condition) and another group that recognizes the robots' presence, which may positively or negatively impact the emotion expressions.

Our results obtained for the *social* condition both from the arousal-valence and the action unit detection confirm high involvement of positively connoted emotions but with varying amplitudes in the expression level. More interesting is the fact that our analysis of the temporal profiles reveals mostly stable and positive arousal patterns over the whole time course of the experiment and an increase of positive valence *after* and beyond the task onset. Similarly, we observed mostly activation of positively connoted action units, which are also persistent over the experiment time, in contrast to the other two conditions where the peak pattern of expressions often coincides with a task onset. We believe that this result shows that the socially-behaving robot positively impacts the experiment, as we have shown signs of frustration or anger for the *non-social* condition. Here, we might see the positive impact of a match between a humanoid appearance of the robot with its social behaviors which the participants can more easily attribute the role of a social companion to. The presence can also create a more social environment where the participants may also feel more comfortable and do not only focus on the task and on possible mistakes in their task performance. We also think that the results show a separation of participants into introvert and extrovert humans. Additionally, and more interesting, is that our inter-subject analysis reveals differences in amplitudes both for arousal-valence and AU facial expressions in all three conditions. As this result is obtained independent of the specific robot condition, it confirms the significance of the human personality in HRI studies.

## 7. Discussion and Limitations of Affective State Recognition

In this study, we were interested to identify how affective behavior changes when humans are exposed to a humanoid shaped robot with or without socially convenient behaviors while performing a sequence of cognitively demanding tasks. We selected the eye gaze, arousal-valence, and activation of action units coding for discrete emotion classes (FACS) as descriptors of the human behaviors because they have been shown to elicit the most information. Additionally, the combined analysis of arousal-valence with action units allowed us to reveal both the emotional responses from the participants' faces and the corresponding amplitudes. This way, we can soften the hard boundaries given by discrete classes like “happy” to more realistic descriptions such as “delightful.” For all three conditions, our results obtained from the arousal-valence distributions aligned well with the detection of facial action units. For our baseline, the *no-robot* condition, we observed a rather flat distribution of emotional expressions toward the neutral or calm state with less pronounced facial activity, especially for action units involved in smiling. In contrast, the *social* condition showed compact clusters toward the positive arousal-valence dimension and higher activations of action units involved in the expression of “happy” states and smiling. The most diversity in the affective state expressions is detected for the *non-social* conditions, covering a wider spectrum of positive and negative arousal-valence responses. Moreover, our analysis demonstrated that more action units were activated and within a wider range of intensity levels. Finally, our temporal analysis showed that participants either concentrate on the task, i.e., peak activations at task onset, or are influenced by the robot during the task, both in positive and negative direction. Due to the delay effects given by the robot talking, we believe that the results confirm a confusion between perceiving a humanoid shaped robot engaging in the task but without social signs. Although the results confirm the influence of an embodied robot on human affective states, we also presented examples of persons displaying affects or facial expressions with low or high amplitudes. Although the *no-robot* condition revealed affective behaviors mostly connected to monotonous human behavior, we could also determine expressive persons. *Vice versa*, the participants in the *social* condition could also be clustered into persons who exhibit, e.g., high arousal and those who were rather calm. Therefore, the mere presence of a socially-behaving robot is not the only essential component in the interaction but also the ability of a robot to adapt to individual human personalities. Our analyses in this study fit squarely into recent research on interaction with social robots which can adapt to a human's personality (de Graaf and Allouch, 2014; Tanevska et al., 2019) and we opt for a better understanding of a robots' personality in the future as suggested by Churamani et al. (2020) in everyday life such as in assistive tasks in domestic environments, driving or tutoring.

In our research, we also target the question of how machine learning is a reliable tool for the automatic extraction of affective states. In the last decade, many approaches primarily based on deep learning rely on pretraining facial images and emotion expressions on large datasets (Baltrusaitis et al., 2018; Barros et al., 2020). The available software seems promising for HRI tasks as it potentially enables researchers from other than ML areas to use those tools to explain higher-level human phenomena. While this approach facilitates HRI research and progress in social robotics, it is a well-known problem that pretrained ML models are heavily biased toward gender, ethnics, and age. Moreover, models of emotions classified into discrete categories as is the case for the action units are trained on scripted, often exaggerated images. However, in HRI experiments it is inevitably important to study the human “in the wild” and to consider the context. Finally, also simple environmental changes like lighting conditions, differences in sensors, or experimental set up can change the output of ML models, which can mislead the interpretation of human emotional behavior. As we are interested in the robustness of the models employed in this study, we also analyzed the software regarding their vulnerability.

### 7.1. Software Limitations Impact the Interpretation of Affective States and Emotions

**Figure 12:**
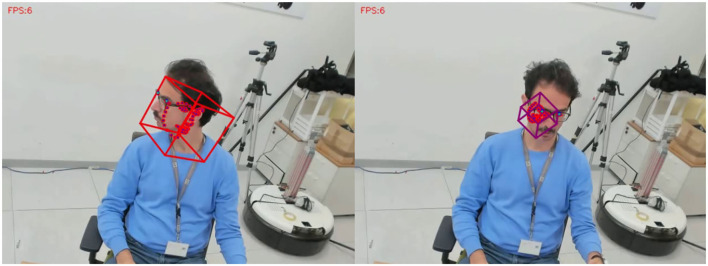
Demonstration of tracking failures due to head movements or insufficient extrapolation of the face or loss of the face template when participants look down. Consequently, an automatized analysis and interpretation of behaviors can be misguided by those outliers.

**Figure 13:**
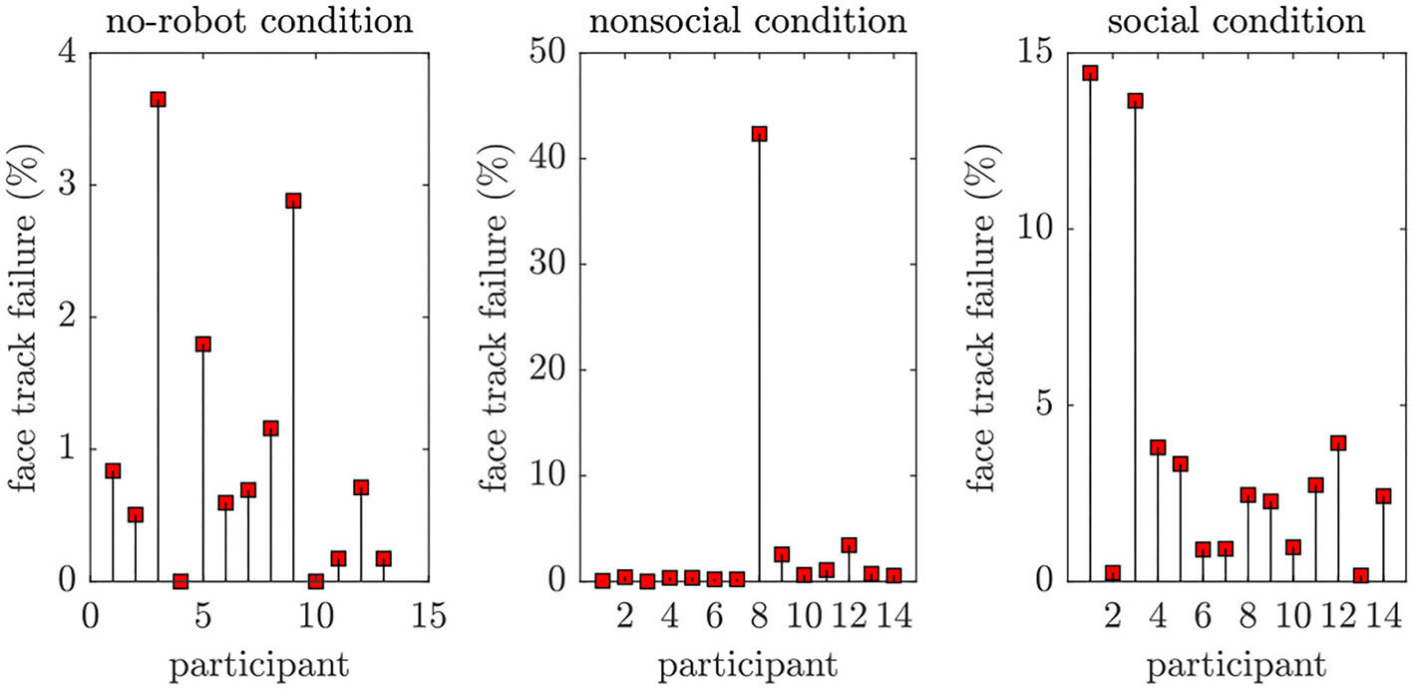
Percentage of incorrectly tracked face (confidence < 0.7) considering all experimental phases (instructions, post-experiment phase). Although we only analyse the concrete experiment start and end phase, the general point here is that tracking failures can occur for more motion-intensive HRI scenarios.

We want to conclude the article with some critical remarks using automatized affective state recognition and their interpretation to sensitize other researchers novel to this field. The diversity of facial expressions and body behaviors such as head movements or gaze put a challenge to every automatic recognition system. Deep learning and access to massive data have revolutionized the way we compute critical features from human behaviors. However, software systems are not trained in a uniform way, i.e., the underlying datasets as well as techniques applied to track a face or to extract action units differ. As a consequence, researchers new to the machine learning field must not treat existing software as a black-box but rather as an additional tool to identify and explain human factors in social robotic applications. We want to underpin some of the important aspects with examples observed in our study. First, we observed face tracking failures revealed by loss or mismatch of the face bounding box as demonstrated in Figure 12. To quantify the impact of such tracking failures over the whole video sequence, we computed: (*#confidence* ≤ 0.7)/*length*_*seq*_, which is the ratio between face tracking failure expressed in a confidence^5^ value below 0.7 and the total length of the video. Figure 13 shows the distribution of such tracking errors over all participants. Although the head movements were reduced during our experiment, the tracking issues can potentially introduce confound effects and thus be misinterpreted as interesting eye gaze or head movement behaviors. The problem may potentiate if more than one person is on the scene.

Second, we address the robustness of the action unit detection. Our analysis demonstrated that overall the intensity levels of action units are on average around 0.5–2 although some participants showed clear expressions of, e.g., “brow lowerer” (AU4, anger). As we combined our analysis with the detection of arousal-valence, we were able to find the separation between people being more introverted vs. the ones showing more extroverted behaviors. However, it was difficult to conclude the co-activation for action units representing the discrete emotion classes. The appearance of single action units in multiple emotion classes (e.g., AU2 is involved in four out of six categories) further hinders a clear interpretation of human emotions. The reasons for intensities on small scales can be a direct consequence of a mismatch between the face data the software was trained and the faces obtained during the experiment. Usually, popular face databases used for training comprise frontal images with persons looking straight into the camera and who are exposed to similar lighting conditions. Different backgrounds and even diversity in gender, age, and ethnics are often not accounted for. Therefore, deriving data from experiments diverging from specific settings can lead to weak AU detections and even incorrect emotion labeling. The failure for the extraction of the action unit AU28 “lip suck” in our data shows the most severe case of AU detection failure. Across all participants and conditions, AU28 was 0 (absent). However, in a *post-hoc* video analysis, we observed some participants showing activity of AU28 during the experiment, especially after an event. AU28 is a relevant feature involved in the characterization of affective states and its omission is critical for the interpretation and claims derived from experiments. Finally, we described the high activation of action unit AU17 (“chin raiser”), which uniquely occurs in the “disgust” class. As we could not find co-activation with other action units from this class, we conclude that AU17, interpreted in isolation, could also be a sign of sadness or surprise. Alternatively, it could have also been potentially confused by the software. Similarly, we observed strong activation of AU25 (“lips part”) which does not appear in any of the emotion classes. From the mouth movement, we conclude that the activity relates to “surprise,” being in accordance with our evaluation of action units.

We also want to point to the absence of other action units like AU46 (“wink”). Although we did not need the whole set of action units, it might be necessary to retrain existent software on data more suitably related to the HRI scenario or consider other variables such as gender and cultural background. The latter aspect is especially important in cross-cultural research as the display of emotions is often guided by social norms.

Finally, we observe a negative influence of lighting condition variations on the classification of arousal and valence as extracted by the FaceChannel (Barros et al., 2020). We changed the image brightness to a darker level to simulate scenarios such as cloudy days, driving in the early evening, or a person moving. We observe a drastic change in the arousal-valence values compared to the original profile, where the valence is more affected than the arousal dimension and shows higher values than for the original images. In the context of the present study, this result shows how small changes in environmental conditions can lead to misinterpretations and false claims derived from the analysis. Although we highlight the limitations perceived during the analysis of this particular experiment, we believe that our discussion generalizes to other scenarios and experimental data, too. While deep learning advances the field of affective state or emotion recognition, the usage of automatization tools in HRI is still in its infancy. For future applications of affective state and emotion recognition systems, it is desirable to establish datasets with a wider diversity of faces from different age ranges and ethnics. Recent research centers around the question of how to reduce known biases, e.g., a recent study by Kara et al. (2021). Also, the cultural context is essential in the correct estimation of affective states and becomes vital in real-world applications: a positive arousal-valence value or detection of the “happy” class due to the high intensity of corresponding action units can indeed signify a real positive state. On the other hand, there are multiple scenarios where humans smile, e.g., to cover an embarrassment or even in tragic situations. The inference “smiling → happy” is the simplest inference, neglecting the vast spectrum of positive emotions. Based on the analysis in this study, we will further analyze the state-of-the-art facial expressions or emotion recognition models (like deep neural networks) evaluated on HRI scenarios for a better understanding of the model requirements in this field and to approach a more realistic classification of human emotions (Barrett et al., 2019).

## 8. Conclusion

In our research, we aim to understand the affective behavior of humans when exposed to cognitively demanding tasks and in presence of a socially-behaving robot. Our analysis of the most crucial features involved in affect expression contrasted between three conditions showed a positive impact on arousal-valence and gaze patterns when humans perform the MATB task with a social robot. In contrast, the presence of a robot with less social skills (*non-social* condition) yielded the most diverse set of affective states and emotional responses with higher amplitudes in the arousal dimension. We believe that the mismatch between the humanoid robot shape with the absence of social cues created the most confusion among the participants and could have potentially elicited stronger emotional responses. Our temporal analysis demonstrated positive or negative affective states also beyond the tasks onsets, which means that the humans get emotionally involved with the robot during the task. However, we can not rule out the role of the humans' personality on certain affective expressions for the three conditions, as we have shown patterns that hint at introvert or extrovert persons. Consequently, we opt to integrate personality traits into affective computing for HRI for a better disentanglement of a persons' natural affective behavior, the human performance of a cognitively demanding task, and the impact of a robot. We also highlight some issues using established software for automatized recognition of affective states and emotion recognition which contributes to a better understanding of to what extent those frameworks can be used for the automatized analysis of human emotional behaviors. The limitations may be considered by other researchers in the field. This way, we hope to inspire future, interdisciplinary work on machine learning for affective state recognition.

## Data Availability Statement

The raw data supporting the conclusions of this article will be made available by the authors, without undue reservation.

## Ethics Statement

The studies involving human participants were reviewed and approved by Liguria Region's Local Ethical Committee in Italy (no. 222REG2015). The patients/participants provided their written informed consent to participate in this study. Written informed consent was obtained from the individual(s) for the publication of any potentially identifiable images or data included in this article.

## Author Contributions

MA ran the experiment and FR helped in the experimental design. DJ implemented the data analysis. DJ and MA discussed the results and wrote the manuscript. All authors contributed to manuscript revision, read, and approved the submitted version.

## Conflict of Interest

MA, TYan, AT, SB, and TYam were employed by the Nissan Motor Corporation. The authors declare that this study received funding from NISSAN. The funder had the following involvement in the study: study design, collection, analysis, interpretation of data, the writing of this article and the decision to submit it for publication.

## Publisher's Note

All claims expressed in this article are solely those of the authors and do not necessarily represent those of their affiliated organizations, or those of the publisher, the editors and the reviewers. Any product that may be evaluated in this article, or claim that may be made by its manufacturer, is not guaranteed or endorsed by the publisher.

## Footnotes

^1^The two tests used in cognitive psychology present congruent and incongruent stimuli; for instance, the word “blue” is written in green color (Stroop). The goal is to test for cognitive skills like selective attention.

^2^We refer to Western European social standards.

^3^For visualization of AUs, see e.g., https://imotions.com/blog/facial-action-coding-system/.

^4^ https://github.com/TadasBaltrusaitis/OpenFace/issues/270

^5^Confidence assigns a value between [0;1], where 0 means loss of the tracked face and 1 notifies successful face tracking.
